# Progress in gasless endoscopic thyroidectomy

**DOI:** 10.3389/fendo.2024.1466837

**Published:** 2024-11-11

**Authors:** Xianbin Cheng, Xiangfu Ding, Sijia Wang, Siyu Li, Hong Zhang

**Affiliations:** ^1^ Department of Thyroid Surgery, The Second Hospital of Jilin University, Changchun, China; ^2^ Department of Burns and Plastic Surgery, Jilin Provincial People’s Hospital, Changchun, China

**Keywords:** gasless, endoscopic, thyroidectomy, approach, advantage

## Abstract

Gasless endoscopic thyroidectomy obviates the necessity for carbon dioxide insufflation to establish a surgical workspace, thus mitigating the potential complications associated with this practice. This technique presents several benefits, such as the maintenance of neck functionality, minimal scarring, and enhanced visibility of the surgical field, which contribute to its extensive adoption in clinical settings. The objective of this study is to synthesize the current methodologies of gasless endoscopic thyroidectomy and to evaluate the advantages and disadvantages inherent to each technique. It aims to offer theoretical insights to assist surgeons in determining the most suitable approach for gasless endoscopic thyroidectomy in their clinical practice.

## 1 Introduction

**Figure 1:**
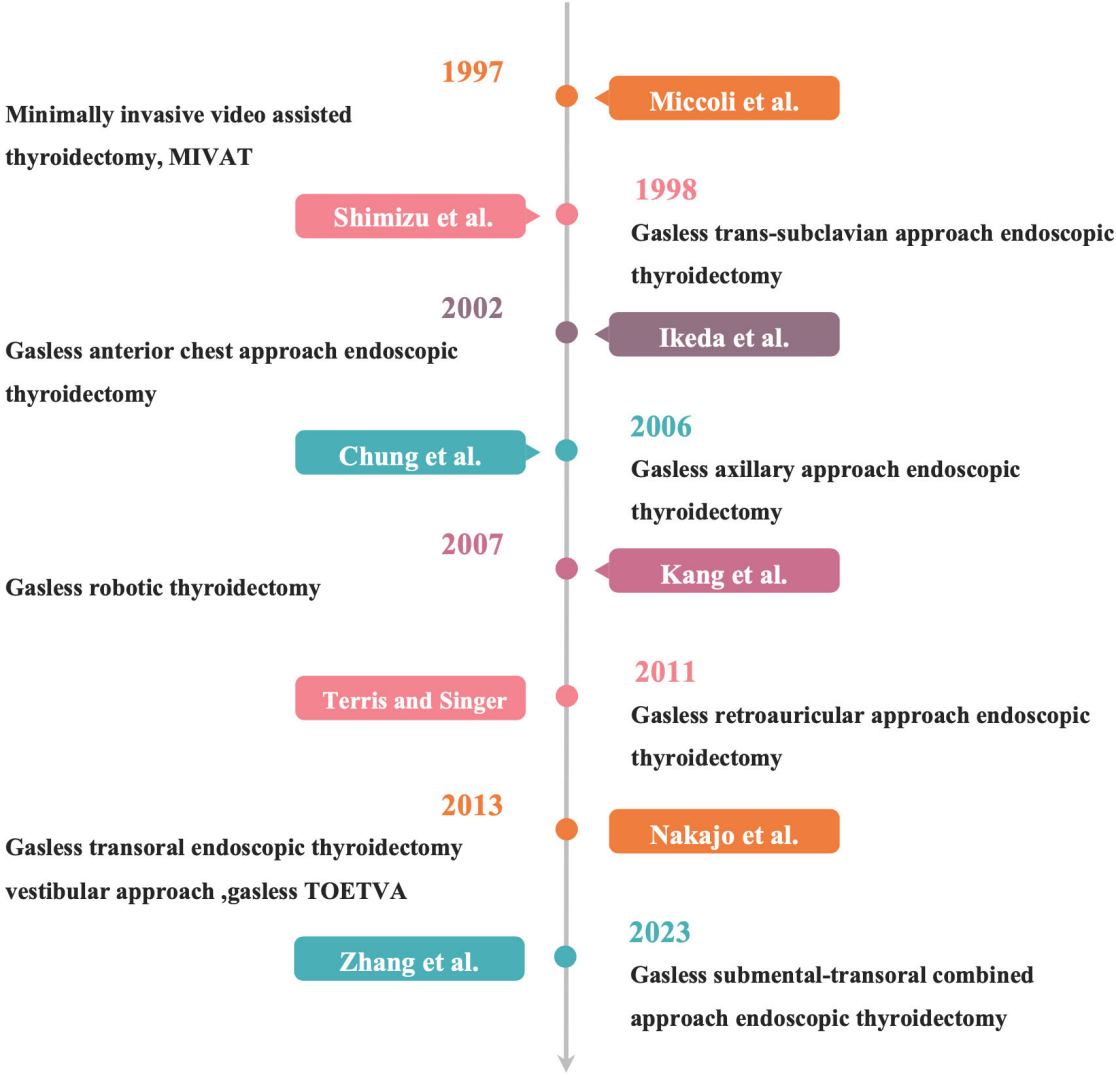
The history of gasless endoscopic thyroidectomy.

Thyroidectomy has been documented for over a millennium (1). Historically, the surgical field during thyroid procedures was compromised by significant hemorrhage due to the rich vascularization surrounding the thyroid gland. A pivotal advancement in thyroidectomy occurred in the late 19th century, when Kocher (2) introduced refined surgical techniques utilizing delicate instruments and the transverse cervical approach. His focus on meticulous dissection aimed to minimize the rates of complications such as hemorrhage, nerve damage, and hypoparathyroidism. In the early 20th century, the development of various endoscopic techniques sought to reduce or eliminate visible cervical scars. Notably, endoscopic parathyroidectomy and thyroidectomy were first reported in 1996 and 1997, respectively (3, 4). However, the lack of natural cavities in the cervical region necessitates the use of CO_2_ insufflation to create a working space for endoscopic procedures, which can lead to complications such as hypercapnia and subcutaneous emphysema. To mitigate these risks, gasless endoscopic thyroidectomy has been introduced, employing a specialized instrument suspension technique to establish an operative space without the need for gas inflation. This approach not only prevents scarring from neck incisions but also diminishes the likelihood of inflation-related complications. The present study aims to review the current state of gasless endoscopic thyroidectomy globally, examining its advancements, benefits, and drawbacks, thereby providing valuable insights for its application in clinical practice (**Figure 1**).

## 2 Gasless endoscopic thyroidectomy

### 2.1 Direction from foot

#### 2.1.1 Minimally invasive video assisted surgery

Professor Miccoli, affiliated with an Italian university, has pioneered a novel approach to non-invasive thyroid surgery. In 1997, he was the first to employ a traditional distractor to facilitate the opening of neck tissue during parathyroid surgery, thereby creating a surgical chamber beneath the strap muscle (5). This entire surgical procedure is executed through a 1.5-centimeter incision located at the sternum of the neck. In 1998, Miccoli (6) extended this technique to the excision of thyroid tumors, achieving an average surgical duration of 87 minutes. This technique is referred to as minimally invasive video-assisted thyroidectomy (MIVAT), also known as classic Miccoli surgery. The postoperative cosmetic outcomes have been reported to be highly favorable, and this surgical approach has demonstrated both safety and efficacy in the resection of thyroid tumors classified as cN0 stage (7).

In 2007, Lombardi et al. (8) implemented a cervical lymph node dissection technique for metastatic papillary thyroid carcinoma, reporting no evidence of residual or recurrent disease in the follow-up of the two patients post-surgery. Subsequently, Chinese researchers Zhang et al. (9) refined the traditional Miccoli technique, enhancing the surgical workspace and instruments. They expanded the permissible length of the incision and employed a specialized suspension device to create the surgical cavity, which significantly improved the operational space. A total of 129 cases of cervical lymph node dissection for metastatic papillary thyroid carcinoma were performed. Statistical analysis of the collected data indicated that this surgical approach is safe, reliable, and effective in achieving thorough excision with a minimal incision, thereby demonstrating clinical applicability.

This technique utilizes a suspension device and does not necessitate CO_2_ inflation, thereby mitigating the risks associated with inflatable endoscopic procedures, such as venous air embolism. Additionally, the incision is considerably smaller than that of traditional open surgery. However, it is noteworthy that this incision resembles the conventional anterior cervical incision, both situated in the readily accessible region of the anterior neck, and does not achieve a completely scarless outcome. The cosmetic results may not be sufficiently pronounced. Furthermore, the requirement for specialized suspension devices to create the surgical cavities results in reduced interstitial space.

#### 2.1.2 Gasless anterior chest approach endoscopic thyroidectomy

In an effort to mitigate the direct visibility of neck incisions, researchers have explored external cervical approaches. In 1998, Shimizu et al. (10) from Japan demonstrated that employing specially designed endoscopic instruments to create a surgical cavity during gasless endoscopic thyroidectomy could address challenges such as limited surgical space and inadequate gas insufflation, which often resulted in suboptimal visualization of the surgical field. The initial report of endoscopic-assisted thyroid surgery via a subclavian approach involved a 4 cm incision beneath the affected clavicle, accompanied by two additional 5 mm incisions in the corresponding neck region. The surgical space was established by utilizing a Kirschner wire to elevate the incision. In 2002, Ikeda et al. (11) modified this technique by making a 3 cm incision below the affected clavicle and two additional 5 mm incisions beneath the sternum and clavicle, ensuring that all incisions remained concealed beneath the clavicle, thereby achieving a scar-free neck.

In 2003, Shimizu’s team (12) applied the subclavian approach for prophylactic cervical lymph node dissection in cases of papillary thyroid carcinoma. This approach involved a lower incision on the anterior chest, allowing for the separation of a substantial neck skin flap. Two Kirschner wires, each 3 mm in diameter, were utilized to suspend the neck skin, creating a cavity, while a 2.0 cm incision was made in the neck for the insertion of an ultrasound knife and forceps. The findings indicated that this surgical method is both safe and feasible for addressing cervical lymph node metastasis in thyroid cancer, offering superior cosmetic outcomes compared to traditional surgical techniques.

In 2011, Huang et al. (13) from China enhanced the gasless transthoracic endoscopic thyroidectomy technique by creating a 30-40 mm skin incision approximately 30-50 mm below the lower edge of the clavicle on the same side of the chest, positioned 50-80 mm from the midline or skin crease of the anterior chest. The incision extended downward to the lower layer of the platysma muscle, with dissection performed under the platysma using a 4 mm diameter, 0° angle endoscope. The surgical plane was established by elevating the skin flap with a retractor, allowing all surgical instruments to be introduced through the incision. A cohort of 219 patients with thyroid disorders underwent this gasless transthoracic endoscopic thyroidectomy, with all procedures successfully completed under endoscopic guidance. Only three patients experienced temporary recurrent laryngeal nerve paralysis, all of whom recovered within 1-3 months postoperatively. Notably, the anterior chest wall approach resulted in no visible neck scars, and patient satisfaction regarding cosmetic outcomes was high.

In 2016, the Huang team (14) conducted a reevaluation of the safety, feasibility, effectiveness, and cosmetic outcomes of gasless anterior chest approach endoscopic thyroidectomy in patients with Graves’ disease. A retrospective analysis of 38 patients revealed an average surgical duration of 87.7 ± 17.3 minutes, intraoperative blood loss of 70.6 ± 11.3 milliliters, and a drainage volume of 42.0 ± 8.5 milliliters. Three cases of temporary recurrent laryngeal nerve paralysis and three instances of temporary hypothyroidism were noted post-surgery, with all patients expressing satisfaction with their cosmetic results. The findings support the assertion that gasless anterior chest approach endoscopic thyroidectomy is a safe, feasible, and effective surgical option for treating Graves’ disease.

In 2022, Guo et al. (15) introduced a modified nipple-areola approach for gasless endoscopic thyroidectomy, utilizing a Kirschner wire adapted from orthopedic surgery to function as a surgical retractor. One end of this retractor was employed to elevate the skin flap, while the other end was connected to a head fixation device. Comparative analysis revealed that CO_2_ levels in the gasless group were significantly lower than those in the gas-insufflation group, which also exhibited notably lower arterial pH levels. Furthermore, the total operation time in the gas-insufflation group was longer, primarily due to the smoke and vapor generated by energy devices obscuring the surgical field, necessitating frequent suctioning and cleaning of the endoscope, which disrupted the procedure and increased intracavitary pressure fluctuations. Consequently, the gasless group demonstrated a significantly shorter surgical duration.

In 2023, a team led by surgeon Zhang (16) employed a specially designed suspension surgical retractor with negative pressure suction to perform neck lymph node dissection for papillary thyroid cancer using an enhanced gasless subclavian endoscopic thyroid surgery technique. The study included 31 patients with papillary thyroid carcinoma, with all surgeries successfully completed under endoscopic guidance and no conversions to open surgery. Four patients experienced temporary hypothyroidism postoperatively, all of whom recovered within one month. Recurrent laryngeal nerve injury is divided into temporary and permanent types. If vocal cord paralysis is confirmed by laryngoscopy after more than 6 months, it is considered permanent recurrent laryngeal nerve injury. Complications associated with cervical lymph node dissection included one case of chylous leakage, which was managed conservatively, and one instance of Horner’s syndrome, which resolved within three months. Throughout the follow-up period, no tumor residue or recurrence was detected. The improved gasless subclavian endoscopic cervical lymph node dissection for papillary thyroid carcinoma demonstrated feasibility, thorough surgical clearance, effective incision concealment, and significant clinical applicability.

Gasless anterior chest approach endoscopic thyroidectomy is recognized as a safe and feasible surgical technique. Currently, this approach encompasses both the subclavian and anterior thoracic areola methods. This technology offers enhanced cosmetic outcomes and presents a novel treatment option for patients with benign tumors, as well as effective alternative treatments for select cancer patients. The subclavian approach preserves the integrity of the cervical white line and protects the anterior cervical structures, significantly reducing postoperative numbness and swallowing discomfort in the anterior cervical region. However, this approach merely relocates the neck incision to the subclavian area, resulting in substantial scars beneath the clavicle, indicating potential for further improvement in cosmetic results. The gasless endoscopic thyroidectomy via the areola approach may encounter challenges in cavity construction efficiency due to the considerable distance from the areola to the thyroid channel, which can complicate the operation and hinder specimen removal due to channel constriction. Additionally, prolonged traction with the cavity-building surgical retractor may increase the risk of skin flap trauma.

#### 2.1.3 Gasless axillary approach endoscopic thyroidectomy

In the year 2000, Ikeda and colleagues (17) were the first to introduce axillary endoscopic thyroid surgery. This technique involved the creation of a 30mm incision in the axilla, through which a 12mm trocar was inserted to facilitate the introduction of an endoscope. Additionally, two 5mm incisions were made in proximity to serve as working ports for surgical instruments. Carbon dioxide (CO_2_) gas was insufflated to maintain a surgical space pressure of 4mmHg; however, this practice occasionally resulted in obscured visibility within the surgical field and potential CO_2_-related complications.

The procedure begins with blunt dissection from the fascial surface of the pectoralis major muscle to the interval beneath the platysma. Dissection continues along the natural plane between the anterior edge of the sternocleidomastoid muscle (SCM) and the sternohyoid muscle. Subsequently, access is gained between the sternohyoid and sternothyroid muscles, followed by the use of an ultrasonic scalpel to divide the sternothyroid muscle, thereby exposing the thyroid gland and establishing the surgical space. Thyroidectomy is then performed in a manner analogous to open surgical techniques (18). Nevertheless, CO_2_ insufflation can lead to a misting effect that compromises the surgical field and may result in complications associated with carbon dioxide.

To mitigate the complications associated with CO_2_ insufflation, Professor Chung and colleagues (19) introduced external traction as an alternative in 2004, aiming to create a more stable surgical environment. This technique involves a 5-6 cm incision in the axilla to form a subcutaneous tunnel extending to the clavicle. A specially designed longer surgical retractor is then inserted beneath the skin flap to elevate the skin, subcutaneous tissue, and platysma muscle. This method provides the advantage of a clear surgical field devoid of gas mist, allowing surgeons to utilize conventional instruments for flap dissection and hemostasis under direct visualization (20, 21).

Initially, the gasless unilateral axillary approach (GUA) for endoscopic thyroidectomy was designed to expose the thyroid gland by dissecting the space between the medial border of the sternocleidomastoid muscle and the lateral border of the strap muscle. Through ongoing refinement by various researchers, more appropriate anatomical pathways and layers have been identified. Since 2006, the approach has evolved to access the thyroid gland through the natural gap between the manubrium and clavicle of the sternocleidomastoid muscle branch, as well as beneath the pretracheal strap muscle (22). This refined technique avoids traditional dissection of the deeper layers of the platysma muscle and eliminates the necessity of separating the strap muscles. The procedure involves accessing the thyroid gland through the space between the manubrium and clavicle, utilizing a surgical retractor to elevate the manubrium of the SCM and liberating the deep surface of the pretracheal strap muscle for endoscopic thyroidectomy. This enhanced approach retains the benefits of the axillary route while ensuring anatomical dissection at the deeper level of the pretracheal strap muscle, thereby preserving the fibrofatty tissue between the deep fascia of the platysma muscle and the pretracheal strap muscle. Compared to open surgery, this method significantly reduces postoperative occurrences of sensory abnormalities in the lower neck and skin tension during swallowing (23).

In 2009, a study conducted by Japanese scholar Kang et al. (24) involved 581 patients with thyroid tumors who underwent gasless endoscopic thyroidectomy via the axillary approach over a decade. None of the procedures necessitated conversion to open surgery. The mean operative time for benign tumors was recorded at 129.4 ± 51.3 minutes, with a postoperative hospital stay averaging 3.3 ± 1.7 days. For malignant tumors, the mean operative time was 135.5 ± 47 minutes, with a postoperative hospital stay of 3.4 ± 0.9 days. Postoperatively, 19 patients experienced transient hypocalcemia, 13 patients exhibited temporary hoarseness, and two patients suffered from permanent vocal cord paralysis. The incidence of postoperative complications was comparable to that observed in open surgery, while the cosmetic outcomes were notably favorable.

A study published in 2020 by Zheng et al. (25) compared 334 patients who underwent unilateral gasless axillary endoscopic thyroidectomy (endoscopic group) with those who underwent traditional unilateral open thyroidectomy (open group). The findings indicated no significant differences between the two groups across nearly all safety indicators, including the number of central compartment lymph node dissections, recurrent laryngeal nerve injuries, surgical blood loss, postoperative bleeding, and infections (p>0.05). The study concluded that gasless axillary endoscopic thyroidectomy is a feasible, safe, and highly satisfactory surgical approach in terms of incision outcomes.

In 2024, Xu et al. (26) conducted a prospective observational longitudinal cohort study involving 134 female patients diagnosed with differentiated thyroid cancer. This study compared the efficacy and quality of life between gasless endoscopic thyroidectomy via the axillary approach (ET) and traditional open thyroidectomy (OT). Various scales were employed to assess differences in quality of life, effectiveness, and safety between the two groups before and after surgery. The results indicated that 68 patients underwent ET, while 66 underwent OT. Compared to the OT group, the ET group demonstrated significant improvements in postoperative physical quality of life, including voice (p=0.036), swallowing (p<0.001), and neck function (p=0.010). Furthermore, the ET group reported significantly higher levels of cosmetic satisfaction (p<0.001) and a more rapid recovery of psychological and emotional well-being. Gasless endoscopic thyroidectomy via the axillary approach thus exhibits favorable cosmetic and psychological outcomes, enhancing postoperative quality of life and facilitating swift recovery for reintegration into daily and social activities.

### 2.2 Direction from head

#### 2.2.1 Gasless transoral endoscopic thyroidectomy vestibular approach

Transoral endoscopic thyroidectomy (TOET), first introduced in 2007 by the K. Witzel team from Austria, has gained popularity among female patients due to its scarless technique (27). Anuwong (28) subsequently enhanced the original method, proposing the transoral endoscopic thyroidectomy vestibular approach (TOETVA). This technique exhibits a complication rate comparable to traditional surgical methods while offering superior cosmetic outcomes. The introduction of TOETVA is considered a significant advancement in surgical practice. Nonetheless, the procedure carries inherent risks, including an increased likelihood of gas embolism resulting from the oral mucosa’s extensive vascular supply following CO_2_ insufflation, as well as potential damage to the sublingual nerve. The advent of gasless transoral endoscopic thyroidectomy vestibular approach (gasless TOETVA) has effectively mitigated these issues associated with conventional endoscopic thyroid surgeries. This method not only protects the sublingual nerve but also eliminates complications related to CO_2_ insufflation (1).

In 2013, Nakajo (29) from Japan was the first to report on gasless TOETVA. The primary technique involves an incision in the vestibular area beneath the lower lip, followed by the creation of a subplatysmal tunnel extending from the vestibule to the anterior neck region, utilizing Kocher’s needle and a mechanical retraction system to elevate the neck skin. The entire procedure is performed through a single channel, providing excellent visualization throughout the operation, which facilitates safe thyroidectomy and central lymph node dissection.

In 2019, Park et al. (30) from Korea developed a novel retractable blade device designed to create and maintain adequate working space for gasless TOETVA. This method employs a three-port technique supported by a self-retaining retractor. The approach was successfully implemented in 15 patients, including 13 cases of thyroid lobectomy and 2 cases of total thyroidectomy. Gasless TOETVA offers ample working space and excellent visibility for thyroid surgery, thereby reducing the risks associated with CO_2_ gas-related complications.

In 2020, Yang et al. (31) conducted an anatomical study to delineate a safe zone for gasless transoral thyroidectomy. This zone is defined by specific anatomical landmarks to ensure safe access to the thyroid gland, thereby minimizing the risk of injury to the mental nerve and marginal mandibular branch. Clinical comparative analyses indicate that gasless transoral thyroidectomy performed within this defined safety zone is a safe and feasible surgical approach.

In 2022, Zhang et al. (32) from China enhanced the complex suspension system for gasless TOETVA. They utilized self-developed retractors, sterile surgical bandages, and anesthesia frames to successfully conduct thyroid surgeries in 75 cases. The average operation time was 143.27 ± 34.60 minutes, with an average retrieval of 8.00 ± 5.39 lymph nodes per patient. Postoperative recovery was favorable, leading the authors to conclude that their innovative gasless TOETVA workspace suspension system provided a stable working environment, improved surgical field clarity, and avoided CO_2_-related complications.

In 2023, Zhang et al. (33) further refined this technique by integrating gasless transoral and submental endoscopic thyroid surgery. They utilized the submental region as the primary operative site, thereby shortening the surgical path and simplifying the procedure, which resulted in significantly reduced operation times. The incision beneath the chin was small and discreet, preserving postoperative aesthetics. By relocating the observation ports to concealed submental areas, they established two operative ports within the oral vestibule. This approach effectively balanced cosmetic outcomes while reducing the “chopstick effect” associated with a single submental incision. Additionally, it lowered infection risks, with patients demonstrating favorable postoperative recovery. Compared to fully gasless transoral vestibular approaches, the combined gasless transoral and submental endoscopic thyroid surgery exhibited slightly inferior cosmetic results.

The gasless TOETVA technique offers the distinct advantage of leaving no visible scars on the skin surface, thereby ensuring optimal cosmetic results. It avoids CO_2_ insufflation, which reduces the risk of air embolism complications. However, certain limitations must be acknowledged: the operating space is relatively confined, which may restrict surgical flexibility and precision. Furthermore, transitioning the surgical incision from a clean to a relatively contaminated site may elevate the risk of postoperative infection. There is also a potential risk of injury to the mental nerve and marginal mandibular branch.

#### 2.2.2 Gasless retroauricular approach endoscopic thyroidectomy

The retroauricular approach to thyroid surgery was first documented by Terries and Singer in 2011 (34, 35). The primary incision is strategically placed in the crease behind the ear, extending beneath the ear and along the occipital hairline, descending approximately 1 cm to ensure complete concealment. This surgical technique employs a gasless robotic approach, initially developed to minimize nerve and vascular damage compared to the axillary approach. The retroauricular route presents unique advantages, including a shorter path relative to the axillary approach, which reduces flap mobilization by 38% (34). Additionally, it alleviates the swallowing discomfort and foreign body sensation often associated with anterior neck approaches, effectively preventing brachial plexus injury.

In 2015, Dr. Lee from Korea (36) reported on thyroid surgery utilizing endoscopic instruments via a retroauricular approach. The study encompassed 47 patients, with an average total operation time of 152 ± 48 minutes and an average flap formation time of 75 ± 24 minutes. The mean endoscopic operation time was 58 ± 18 minutes. Patients undergoing retroauricular thyroidectomy experienced longer operation times compared to the control group (65.21 ± 26.86 minutes for open surgery, p<0.001). Importantly, there were no accidental injuries to the trachea, esophagus, or larynx in either group.

In 2023, Dr. Yulian from Indonesia (37) conducted an early retrospective analysis involving 31 patients who underwent gasless retroauricular approach endoscopic thyroidectomy. The average operation time was 154.2 ± 21.3 minutes, with an average blood loss of 69.2 ± 52.1 milliliters, and an average hospital stay of 4.7 ± 2.2 days. All observed complications were temporary, and all patients maintained good health throughout the follow-up period. A majority of patients (65.6%) expressed high satisfaction with the scar concealed behind the ear. The gasless retroauricular approach endoscopic thyroidectomy demonstrates a safe and viable minimally invasive surgical option, yielding favorable postoperative outcomes.

The gasless retroauricular approach endoscopic thyroidectomy does not require CO_2_ insufflation and utilizes a specialized retractor to establish the operative space, akin to other gasless techniques. This method circumvents the potential complications associated with CO_2_ insufflation, allowing for direct visualization and meticulous dissection of the great auricular nerve, thereby minimizing the risk of nerve damage. The gasless retroauricular approach offers distinct advantages in lymph node clearance in the lateral neck and VI region, particularly beneficial for patients with shorter neck lengths. However, certain limitations exist. For instance, the retroauricular flap tends to be thinner, and continuous retractor traction may lead to subcutaneous bruising. Additionally, inadequate exposure of the contralateral side of the thyroid may pose significant challenges in achieving total thyroidectomy. Furthermore, the approach complicates adequate exposure and management of the upper pole of the thyroid and the anterior neck region, potentially necessitating transection of part of the strap muscles to enhance visibility. Patients with sparse hair may also experience some visibility of the scar at the retroauricular incision site.

### 2.3 Gasless robot thyroidectomy

Since its FDA approval in 2000, the da Vinci surgical robot has been extensively utilized for robot-assisted solid tumor resections across various surgical disciplines. Originally developed for remote treatment of war casualties, robotic surgery has gained widespread acceptance in hospital settings for disease management.

In 2007, Kang et al. (38) performed the first gasless robotic thyroid surgery via the axillary approach, employing an external traction device and four robotic arms to maintain the surgical workspace without CO_2_ insufflation. The authors compared gasless robotic thyroidectomy with traditional thyroid surgery and found no significant differences in intraoperative blood loss, number of lymph nodes cleared, or length of hospital stay. However, the procedure was associated with longer operation times and higher costs. Although incisions under gasless conditions in the axilla are larger than those under gas conditions, the axillary location is less exposed and more concealed, resulting in superior cosmetic outcomes.

In 2017, Kim et al. (39) conducted a comprehensive retrospective study at Yonsei University in South Korea involving 5,000 patients with thyroid tumors who underwent gasless transaxillary approach robot-assisted thyroid surgery. The average operating time was 134.5 ± 122.0 minutes. The postoperative complication rate was 24.1%, with no severe complications reported, and the incidence of complications demonstrated a declining trend over the study period. There were no fatalities during the follow-up period, and 26 cases (0.5%) experienced local recurrence. The study concluded that robot-assisted thyroid surgery via the transaxillary approach is safe and reliable, with perioperative complication rates comparable to those of open surgery.

The retroauricular approach (RA) without gas involves an incision behind the ear at the hairline, similar to parotid gland surgery. This method is more readily accepted by head and neck surgeons. The RA approach features a shorter distance for flap detachment and does not require insufflation. Hyung et al. (40) successfully completed 87 cases of robot-assisted thyroid surgery using the gasless RA approach, with no significant intraoperative complications or conversions to open surgery. Patients reported high satisfaction with the postoperative cosmetic outcomes. However, the RA approach may present challenges for bilateral thyroid lobectomy and clearance due to its top-to-bottom surgical view and sequence, which differs from traditional open surgery. This necessitates adjustments and adaptations by the surgical team, thereby increasing surgical complexity.

In 2019, SHIN I B et al. (41) conducted a study on gasless bilateral axillo-breast approach (BABA) robotic thyroidectomy. They employed a 1-0 polydioxanone (PDS) suture to elevate a flap, securing the junctions of the anterior borders of both sternocleidomastoid muscles to points on diagonal lines extending from the bilateral axillae to the thyroid cartilage. Additionally, they sutured the junctions of the superior borders of both clavicles to points on diagonal lines running from the bilateral nipples to the cricoid cartilage. These four sutures were affixed to Omni-Tract retractors to maintain the surgical working space. The findings concluded that compared to CO_2_ insufflation robotic surgery, gasless BABA robotic thyroidectomy offers advantages such as precise operation, lower risk of metabolic disturbances, reduced postoperative pain, clear visibility of the surgical field, and stable maintenance of operative space.

In 2023, researchers from Europe and Asia published studies on the learning curves associated with gasless robotic thyroidectomy, revealing notable differences. Materazzi et al. (42) employed Cumulative Sum (CUSUM) analysis to assess the learning curve for gasless robotic-assisted axillary thyroidectomy at a tertiary European institution, involving 583 patients. Their findings suggested that for surgeons inexperienced in robotic procedures, mastering robotic axillary thyroid lobe resection required approximately 66 cases, with an additional 56 cases needed to achieve proficiency in robotic total thyroidectomy via the axillary approach. Conversely, Xu et al. (43) studied 105 patients undergoing gasless robotic-assisted axillary thyroidectomy in Asia, determining that for surgeons without prior experience in endoscopic thyroid surgery, the learning curve for this procedure was approximately 31 cases. CUSUM analysis and moving average plots highlighted a distinct transition phase occurring between 25 and 30 cases. The disparities between Western and Eastern studies may be attributed to variations in surgeon experience and patient anatomical factors. Structured training programs could potentially mitigate the steepness of the learning curve. Furthermore, following the learning curve, there was a notable reduction in both surgical duration and complication rates.

## 3 Advantages and drawbacks of gasless endoscopic thyroidectomy

### 3.1 Comparative analysis: gasless vs. gas endoscopic thyroidectomy

Gasless endoscopic thyroidectomy presents several advantages over its gas-assisted counterpart, particularly in terms of reducing complications such as gas embolism and subcutaneous emphysema. This technique also mitigates the risks associated with CO_2_ insufflation, including hypercapnia and hemodynamic fluctuations, thereby decreasing the associated risks of anesthesia management.

Furthermore, gasless surgery allows for the efficient removal of smoke from the surgical field, which diminishes the frequency of lens cleaning and ensures optimal visibility throughout the procedure. This efficiency contributes to simplified surgical techniques and shorter operation times (44, 45). The gasless approach eliminates the need for gas insufflation machines, high-purity CO_2_, and CO_2_ monitoring devices, resulting in substantial cost savings for patients.

By utilizing access routes through the axilla or subclavian region, this method capitalizes on natural muscle gaps in the neck to create a surgical cavity, thereby exposing the thyroid gland situated deep to the strap muscles of the anterior neck. This technique avoids the necessity for anterior neck skin flap separation and midline incisions, thereby preserving the functional integrity of the anterior neck while maintaining aesthetic appearance and sensory and motor functions. Postoperatively, patients typically report an enhanced quality of life, experiencing less discomfort in the suprasternal notch and avoiding complications such as skin-trachea tethering during swallowing (46).

In selecting between gasless and gas endoscopic thyroidectomy, surgeons must consider a range of factors, including surgical objectives, the patient’s medical condition, the surgeon’s expertise, postoperative management, and the availability of medical resources. A comprehensive evaluation is essential to ensure that the selected surgical approach is both safe and effective, ultimately leading to optimal surgical outcomes. For instance, patients with pre-existing cardiovascular or pulmonary conditions may find gasless endoscopic thyroidectomy to be a more appropriate option to reduce the risk of gas-related complications.

### 3.2 The differences between gasless endoscopic thyroidectomy approaches

**Figure 2:**
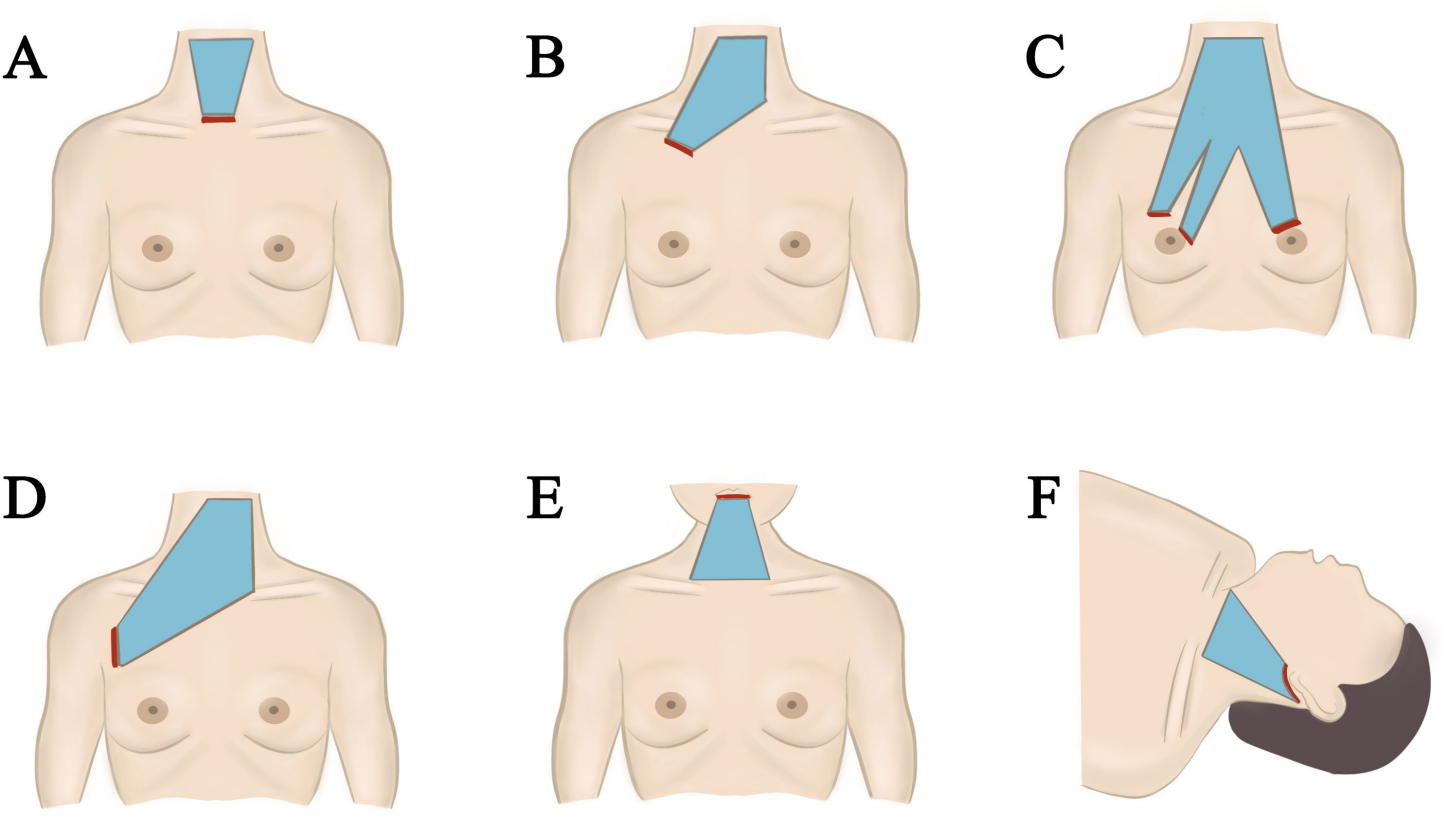
Different approach of gasless endoscopic thyroidectomy. **(A)** Minimally invasive video assisted thyroidectomy. **(B)** Gasless trans-subclavian approach endoscopic thyroidectomy. **(C)** Gasless anterior chest approach endoscopic thyroidectomy. **(D)** Gasless axillary approach endoscopic thyroidectomy. **(E)** Gasless transoral endoscopic thyroidectomy vestibular approach. **(F)** Gasless retroauricular approach endoscopic thyroidectomy.

Both gasless endoscopic thyroidectomy and robotic surgeries primarily create operational space through traction, necessitating the use of specialized suspension instruments regardless of the chosen technique (47, 48). These instruments, which include Kocher clamps and custom hooks, currently lack standardization. Variations in suspension instruments can lead to differing operational spaces for the same surgical approach. Prolonged traction may result in complications such as skin ischemia, subcutaneous hematoma, and noticeable postoperative edema, although these occurrences are rare and typically resolve shortly after surgery (**Figure 2**).

While both gasless endoscopic thyroidectomy and robotic surgeries reduce neck scarring associated with traditional open procedures, the choice of approach still influences cosmetic outcomes. The transoral vestibular approach is regarded as the most aesthetically favorable, classified under Natural Orifice Transluminal Endoscopic Surgery (NOTES) (49). Other approaches yield surgical scars of varying lengths; postauricular approaches can be concealed by hair, axillary approaches are relatively discreet, and anterior chest approaches can be covered by clothing (50).

**Figure 3:**
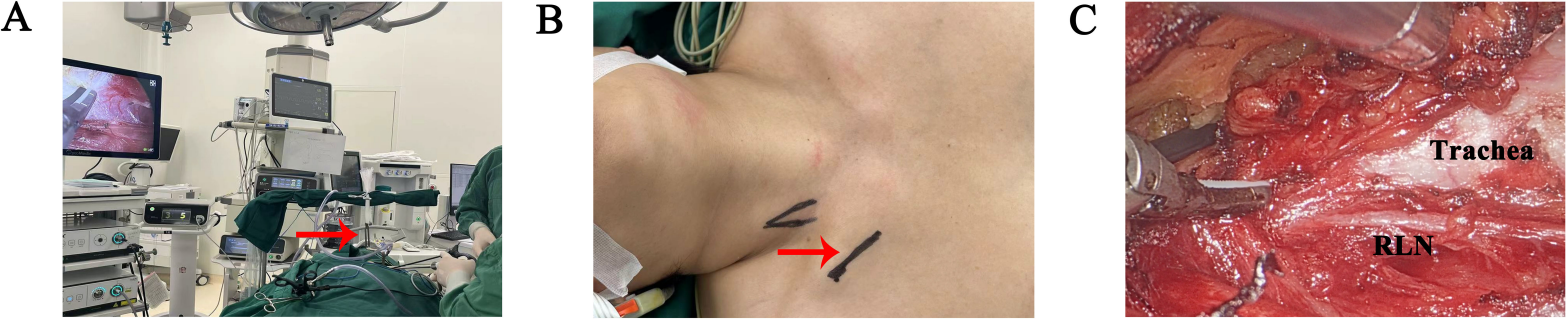
Surgical procedures of gasless trans-subclavian approach endoscopic thyroidectomy at the second hospital of Jilin University. **(A)** Overall surgical scene (red arrowhead: surgical retractor). **(B)** Incision design (red arrowhead). **(C)** Intraoperative observation, after thyroid separation. (RLN recurrence laryngeal nerve).

Among patients undergoing thyroidectomy at our institution, unmarried women often prefer scarless transoral vestibular surgeries, followed by axillary and subclavian approaches. Cultural considerations lead some female patients to prioritize breast preservation and to oppose anterior chest approaches. Concurrently, some patients may express apprehension regarding transoral vestibular approaches, necessitating thorough preoperative counseling by healthcare providers (**Figure 3**).

In terms of total surgical duration, non-inflatable endoscopic surgeries primarily allocate time to flap dissection. The transoral vestibular approach presents particular challenges due to the need to navigate the surface of the mandible. Axillary and periareolar approaches require longer incisions to access the thyroid gland. Consequently, postauricular, subclavian, and MIVAT approaches tend to have shorter incisions and operation times.

Regarding flap dissection range, the axillary approach encompasses the widest range among all procedures (51). However, it also carries a higher risk of damaging subcutaneous vascular nerves during dissection. Conversely, MIVAT and transoral vestibular approaches involve minimal flap dissection, but they also provide smaller operating spaces, exacerbating the “chopstick effect,” which is particularly pronounced during retroauricular and transoral vestibular approaches due to the thin, broad, and fragile nature of the neck flap fat tissue, which is susceptible to skin damage.

For patients undergoing bilateral thyroidectomy, the retroauricular, axillary, and anterior chest (subclavian) approaches present greater challenges for contralateral thyroidectomy and lymph node clearance. The transoral vestibular approach excels in clearing VI area lymph nodes but is less effective in achieving thorough clearance of lateral neck lymph nodes.

With respect to robotic surgery, patient preferences for surgical approaches vary based on differing economic conditions and insurance coverage across countries. Under similar circumstances, the transaxillary thyroidectomy option provides surgeons with improved visibility, a more relaxed operational state, and greater stability during robotic procedures. Consequently, robotic surgery is increasingly favored by surgeons (52). However, it necessitates a longer learning curve compared to endoscopic thyroidectomy. Once surgeons attain proficiency in various surgical approaches, they should prioritize patient-centered choices when determining the most appropriate surgical method based on specific surgical requirements (53).

### 3.3 Drawbacks of gasless endoscopic thyroidectomy

While gasless endoscopic thyroidectomy offers significant benefits over traditional open surgery, including minimally invasive techniques, expedited postoperative recovery, and reduced intraoperative bleeding, it is not without its challenges. The emergence of nonconventional complications poses significant hurdles for gasless endoscopic thyroidectomy. Rossi et al. (54, 55) conducted a study involving 541 patients who underwent robot-assisted trans-axillary thyroidectomy (RATT). In this study, one patient experienced brachial plexus nerve injury, resulting in functional impairment of the distal branch of the ulnar nerve. Another patient reported the occurrence of seeding of a benign goiter, while four patients developed seroma. A small number of patients who underwent transoral endoscopic thyroidectomy via vestibular approach (TOETVA) experienced skin hematomas and chin numbness (56). Fortunately, the incidence of these nonconventional complications remains relatively low. Although gasless endoscopic thyroidectomy carries potential risks associated with the introduction of new techniques, such occurrences are infrequent. It is imperative for surgeons to remain vigilant regarding the potential for these complications and to be knowledgeable about preventive measures.

## 4 Conclusion

Gasless endoscopic thyroidectomy, a surgical intervention for thyroid tumors, has gained significant traction in clinical practice. Various approaches, including axillary, anterior chest, transoral, postauricular, and their combinations, provide multiple options for patients with thyroid tumors. This technique employs specialized retraction devices to maintain the surgical space without the need for gas inflation, thereby mitigating complications associated with CO_2_ insufflation. The advantages of gasless endoscopic thyroidectomy include effective tumor excision, preservation of neck functionality, inconspicuous and aesthetically favorable incisions, and enhanced visibility during the procedure. Nonetheless, the acquisition of proficiency in this technique presents certain challenges, necessitating substantial experience with a sufficient volume of cases and the navigation of a learning curve to achieve optimal therapeutic and cosmetic results. This study focuses exclusively on gasless endoscopic thyroidectomy, which represents a limited subset of the diverse surgical options available for thyroidectomy. In the selection of a surgical approach, it is imperative that the primary emphasis is placed on effective treatment, followed by the preservation of function and the attainment of satisfactory cosmetic outcomes.
